# A Multi-Step Chromatographic Approach to Purify Radically Generated Ferulate Oligomers Reveals Naturally Occurring 5-5/8-8(Cyclic)-, 8-8(Noncyclic)/8-O-4-, and 5-5/8-8(Noncyclic)-Coupled Dehydrotriferulic Acids

**DOI:** 10.3389/fchem.2018.00190

**Published:** 2018-06-08

**Authors:** Martin Waterstraat, Mirko Bunzel

**Affiliations:** Department of Food Chemistry and Phytochemistry, Institute of Applied Biosciences, Karlsruhe Institute of Technology, Karlsruhe, Germany

**Keywords:** ferulate trimers, ferulic acid, phenolic cross-links, plant cell walls, radical coupling

## Abstract

Ferulate-mediated cross-linking of plant cell wall polymers has various implications on the quality of plant based food products, forage digestibility, and biomass utilization. Besides dehydrodiferulic acids (DFA), dehydrotriferulic acids (TriFA) gained increasing interest over the past two decades, because they potentially cross-link up to three polymers. Here, we describe a separation strategy to obtain several TriFA as analytical standard compounds from a reaction mixture after radical coupling of ethyl ferulate. By using silica flash chromatography, Sephadex LH-20 chromatography, and reversed phase HPLC, six known TriFA as well as three previously unidentified ferulic acid trimers were obtained, and their structures were characterized by mass spectrometry and NMR spectroscopy (^1^H, HSQC, COSY, HMBC, and NOESY). The novel trimers were identified as 5-5/8-8(cyclic)-, 8-8(noncyclic)/8-O-4-, and, tentatively, 5-5/8-8(noncyclic)-TriFA. Natural occurrence of these TriFA in plant cell walls was demonstrated by LC-MS/MS analyses of alkaline cell wall hydrolyzates.

## Introduction

Ferulic acid is widely distributed in plant cell walls. Esterified to arabinoxylans, larger amounts of ferulic acid can be found in monocotyledonous plants, particularly within the Poaceae family (Smith and Hartley, 1983; Harris and Trethewey, 2010). Feruloylated arabinan and galactan structural units were also identified in some plants belonging to the Amaranthaceae family, concluding that ferulic acid is esterified to pectins in specific dicotyledonous plants (Ishii and Tobita, 1993; Colquhoun et al., 1994; Habibi et al., 2004; Levigne et al., 2004; Bunzel et al., 2005b; Wefers et al., 2014).

Oxidative conditions caused by cell wall peroxidases/H_2_O_2_ or laccases/O_2_ trigger ferulate radical coupling reactions resulting in the formation of ferulate dehydrodimers, -trimers and -tetramers (Ralph et al., 2004; Bunzel, 2010). Oxidative coupling of polysaccharide bound ferulates generates cross-links between polysaccharides (Saulnier et al., 1999; Allerdings et al., 2005; Ralet et al., 2005; Bunzel et al., 2008a; Wefers and Bunzel, 2015), but also results in linkages to lignin (Lam et al., 1992; Jacquet et al., 1995; Ralph et al., 1995; Bunzel et al., 2004b) and, possibly, cell wall proteins (Piber and Koehler, 2005).

Formation of polymer bound ferulate oligomers affects cell wall properties such as firmness and resistance against biotic and abiotic stress factors as well as enzymatic polysaccharide degradation as a key step of forage digestibility and in biofuel production (Ralph et al., 2004; Grabber et al., 2009). Additionally, generation of ferulate oligomer based cross-links may partially contribute to the crispness of fruit and vegetables (Parker and Waldron, 1995; Parr et al., 1996; Waldron et al., 1997), is responsible for covalent gel formation of pectins (Micard and Thibault, 1999; Oosterveld et al., 2000, 2001), and has an impact on dough and bread quality (Goesaert et al., 2005).

However, analysis of ferulate oligomers in plants and plant based food products is demanding due to the vast variety of regioisomers and their limited availability as standard compounds. Low amounts of dehydrotriferulic acids (TriFA) compared to dehydrodiferulic acids (DFA) require highly sensitive analytical methods (Dobberstein and Bunzel, 2010; Jilek and Bunzel, 2013). Despite their comparably low concentrations in plant cell walls, TriFA theoretically cross-link up to three cell wall polymers, having a considerable impact on cell wall properties (Bunzel, 2010). Therefore, strong efforts were made to characterize TriFA from plant materials and to provide TriFA standard compounds (Bunzel et al., 2003a, 2005a, 2006; Rouau et al., 2003; Funk et al., 2005; Waterstraat et al., 2016). Despite, isolation procedures from natural sources such as maize bran are time-consuming and sparsely suitable for the isolation of low-concentrated TriFA. Targeted synthetic procedures for each regioisomer are extremely time-consuming, too, but also require advanced skills in synthetic organic chemistry (Mouterde et al., 2013).

Here, we describe a convenient procedure to synthesize and purify TriFA as standard compounds. Also, three novel TriFA regioisomers were characterized and detected in mono- and dicotyledonous plant samples for the first time.

## Experimental

### Chemicals

Research chemicals were obtained from the following suppliers: Cu(I)Cl, copper(II)-tetramethylethylenediamine (TMEDA), HCl (37%), NaOH, ammonium chloride, ethyl acetate, and ethanol from Carl Roth GmbH (Karlsruhe, Germany); acetonitrile, diethyl ether, methanol, acetone, petroleum ether, tetrahydrofuran, and Na_2_SO_4_ from VWR International (Radnor, PA, USA); acetone-*d*_6_, ferulic acid, [1-^13^C]triethyl phosphonoacetate, and trifluoroacetic acid from Sigma-Aldrich (St. Louis, MO, USA); D_2_O from deutero GmbH (Kastellaun, Germany); acetyl chloride and vanillin from Fluka (Buchs, Switzerland); formic acid from Merck KGaA (Darmstadt, Germany); NaHCO_3_ from Riedel-de Haën AG (Seelze, Germany); carbogen (5% CO_2_) from Air Liquide S.A. (Düsseldorf, Germany), and NaH (60% dispersion in oil) from Alfa Aesar (Karlsruhe, Germany).

### Plant samples

Wheat grain (*Triticum aestivum* L.) and popcorn maize (*Zea mays* L. var. *everta*) were purchased from a local grocery store in Karlsruhe, Germany. Amaranth (*Amaranthus hypochondriacus* L.) was grown and seeds were harvested in 2012 in Moersingen, Germany. Sugar beet pulp (*Beta vulgaris* L. var. *vulgaris*) was kindly donated by Südzucker AG (Mannheim, Germany) in 2013.

### Synthesis of [9-^13^C]ferulic acid

#### Vanillin acetate

As previously described by Yang et al. (2011), vanillin (3 g, 19.7 mmol) was dissolved in pyridine (5 mL) and acetylated by dropwise addition of acetic anhydride (20 mL) at 0°C. The solution was warmed to room temperature and stirred for another 6 h. The reaction product was extracted into chloroform (3 × 40 mL), and the combined organic phases were washed with H_2_O (4 × 20 mL). The organic solvent was removed by rotary evaporation, yielding vanillin acetate (3.75 g, 19.33 mmol).

#### [9-^13^C]ferulic acid

[9-^13^C]ferulic acid was synthesized based on the procedure of Lu et al. (2010). NaH (60% dispersion in oil, 190 mg, 4.75 mmol) was suspended in tetrahydrofuran (20 mL), and the mixture was cooled to −20°C. While stirring, [1-^13^C]triethyl phosphonoacetate (1 g, 885 μL, 4.46 mmol) was added dropwise resulting in a yellow-white haze. After warming to room temperature and stirring for another 30 min, a clear, yellow solution was obtained. The reaction mixture was cooled to −20°C, and vanillin acetate (970 mg, 5 mmol, dissolved in 2 mL of tetrahydrofuran) was added dropwise. The solution was warmed to room temperature and stirred for another 2 h, before the solvent was removed by rotary evaporation. NaOH (2 M, 30 mL) was added to the residue, and the solution was stirred overnight at 50°C. The reaction products were extracted into ethyl acetate (3 × 30 mL), and the organic solvent was removed by rotary evaporation.

This procedure was repeated twice to obtain 3.2 g of solids, containing primarily [^13^C]ferulic acid and vanillin. The raw material was dissolved in ethyl acetate (12 mL), and [^13^C]ferulic acid was extracted into NaHCO_3_ (5% aqueous solution, 3 × 12 mL). The combined aqueous fraction was washed with ethyl acetate (3 × 12 mL) before the pH was adjusted carefully to 1 using concentrated HCl. Then, [^13^C]ferulic acid was re-extracted into ethyl acetate (3 × 12 mL), and the organic solvent was removed by rotary evaporation, yielding 2.42 g of [9-^13^C]ferulic acid (11.43 mmol, 85.4% yield).

### Synthesis and purification of ferulic acid oligomers

Addition of acetyl chloride (2.5 mL, 2.75 g, 35 mmol) to a solution of ferulic acid (5 g, 25.8 mmol) in 50 mL of ethanol and stirring at room temperature overnight yielded ethyl ferulate. The solvents were evaporated under reduced pressure at 40°C, and the reaction was repeated for quantitative conversion. The residue after evaporation was re-dissolved in ethanol (3 × 10 mL) and evaporated intermediately to remove remaining HCl. The oily product crystallized spontaneously at −20°C overnight.

To generate ferulate oligomers, the catalytic procedure developed by Lu et al. (2012) was used in a slightly modified way as described earlier (Waterstraat et al., 2016). In brief, ethyl ferulate (888 mg, 4 mmol) was added to a solution of CuCl (40 mg, 0.4 mmol) and TMEDA (60 μL, 0.4 mmol) in acetonitrile (200 mL). A balloon filled with carbogen (5% CO_2_ in O_2_) was used to change the gas atmosphere within the two-neck flask and the reaction mixture was stirred vigorously for 4 h at room temperature. The reaction was stopped by adding HCl (1 M, 24 mL), and the concentration of acetonitrile was reduced by rotary evaporation before the reaction products were extracted into ethyl acetate (3 × 40 mL). After washing the combined ethyl acetate fractions with acidic NH_4_Cl solution (2 mL of 1 M HCl plus 20 mL of saturated NH_4_Cl), the organic solvent was removed by rotary evaporation.

The crude product was fractionated by flash chromatography (Biotage Horizon flash chromatograph, Charlottesville, VA, USA) using a prepacked silica column (30 g of Si60, 15–40 μm particle size; Götec-Labortechnik GmbH, Bickenbach, Germany) as described earlier (Waterstraat et al., 2016). Petroleum ether and ethyl acetate were used as elution solvents A and B, respectively, and the following binary elution gradient was applied with a flow rate of ~70 mL/min: initially 30% B, linear increase to 60% B within 1065 mL. Subsequently, the column was eluted with 550 mL of 100% B to obtain a fraction that mostly contained 5-5-coupled dehydrodiferulate and ferulate trimers and tetramers. The organic solvent of this fraction was evaporated, and the residue was saponified by adding NaOH (2 M, 20 mL) protected from light and O_2_ for 18 h at room temperature. After acidification (pH < 2) with concentrated HCl, the hydrolyzed products were extracted into ethyl acetate (3 × 5 mL), and the solvent of the combined extracts was removed under reduced pressure. The residue was re-dissolved in methanol/H_2_O (1:1, v/v, 10 mL), and loaded onto a Sephadex LH-20 column (87 × 2.6 cm, GE Healthcare Biosciences, Pittsburgh, PA, USA) for further fractionation.

**Figure 1:**
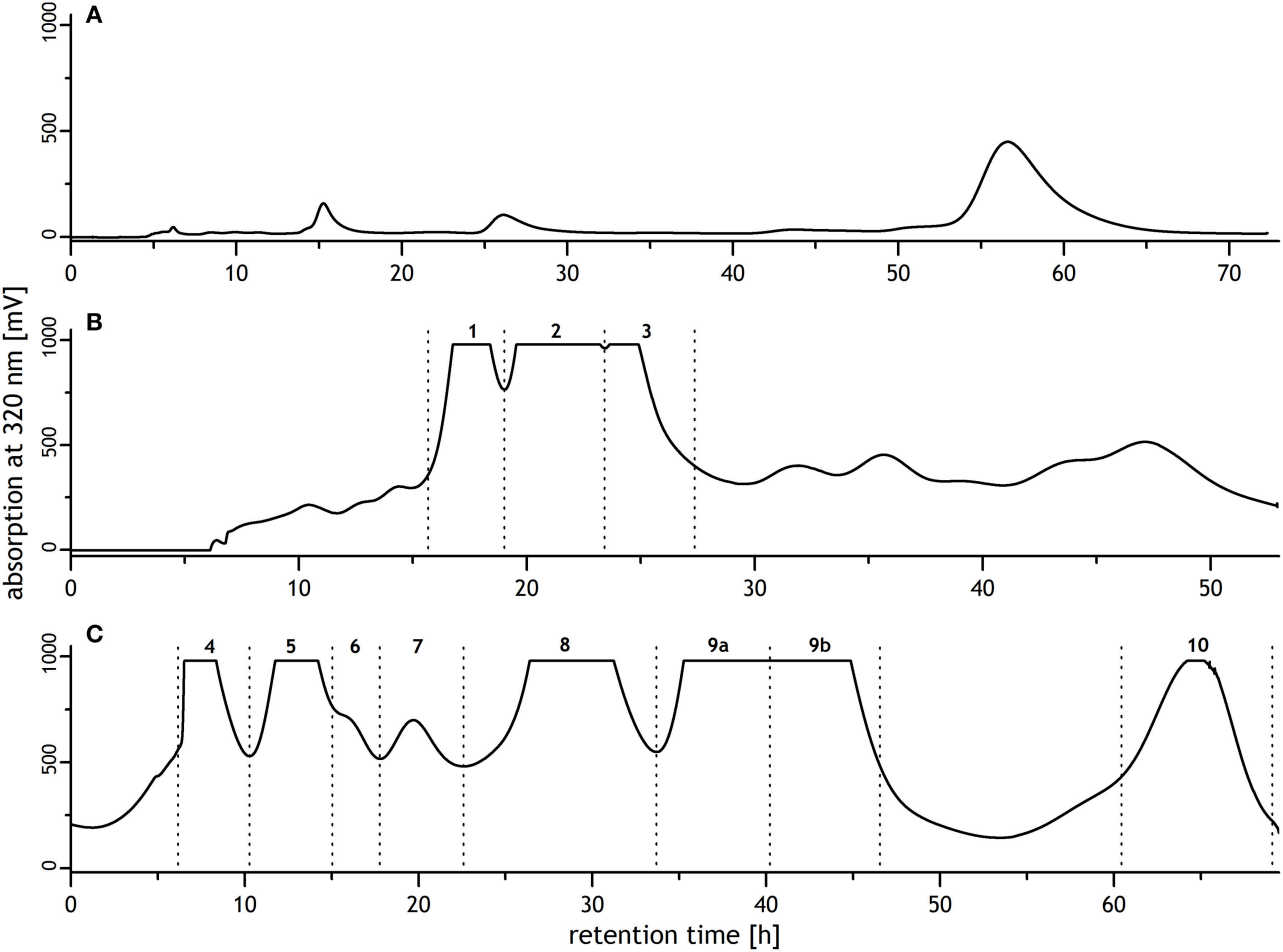
Sephadex LH-20 chromatograms of a mixture of oligoferulic acids generated by radical coupling of ethyl ferulate. Chromatographic conditions were **(A)** 0.5 mM aqueous trifluoroacetic acid/methanol 95/5 (v/v), flow rate: 1.5 mL/min; **(B)** 0.5 mM aqueous trifluoroacetic acid/methanol 50/50 (v/v), flow rate: 1.0 mL/min; **(C)** 0.5 mM aqueous trifluoroacetic acid/methanol 40/60 (v/v), flow rate: 1.0 mL/min. Collected fractions contained: 1, unknown; 2, 8-8(cyclic)-dehydrodiferulic acid (DFA) and trimer **1**; 3, 8-8(noncyclic)-DFA; 4, trimer **2**; 5, 8-8(cyclic)/5-5-dehydrotriferulic acid (TriFA); 6, monoethyl ester of trimer **3**; 7, unknown; 8, trimer **3**; 9, 5-5-DFA and 8-5(noncyclic)/5-5-TriFA; 10, 5-5/8-O-4-TriFA.

Sephadex LH-20 chromatography was performed according to Bunzel et al. (2004a) in three isocratic elution steps. Step 1: elution with 0.5 mM aqueous trifluoroacetic acid/methanol (95:5, v/v); flow rate, 1.5 mL/min; elution time, 73 h. Step 2: 0.5 mM aqueous trifluoroacetic acid/methanol (50:50, v/v); 1.0 mL/min; 53 h. Step 3: 0.5 mM aqueous trifluoroacetic acid/methanol (40:60, v/v); 1.0 mL/min; 70 h. UV detection was carried out at 320 nm, and fractions of 6 mL each were collected automatically. Fractions were combined based on their UV spectra (Figure 1), and fractions 2, 4, 5, 6, 8, 9, and 10 were purified by preparative reversed phase high performance liquid chromatography (RP-HPLC).

Preparative RP-HPLC (two LC-8A pumps, SPD-20A UV-detector, CBM-20A communication bus module, Shimadzu, Kyoto, Japan) was performed using a C18 Luna column (250 × 15 mm, 5 μm, 100 Å; Phenomenex Ltd., Aschaffenburg, Germany). UV detection was performed at 320 nm, and the following binary gradients of water containing 0.01% formic acid (A) and either acetonitrile containing 0.01% formic acid (B) or methanol containing 0.01% formic acid (C) were used (flow rate of 8 mL/min, room temperature). Fraction 2: initially 25% C, linear increase to 50% C within 20 min, followed by rinsing and equilibration steps. Trimer **1** (8-[5-[(1*E*)-2-carboxyeth-1-en-1-yl]-2-hydroxy-3-methoxy-phenyl]-7-hydroxy-1-(4-hydroxy-3-methoxyphenyl)-6-methoxy-1,2-dihydronaphthalene-2,3-dicarboxylic acid) eluted after 20 min. Fraction 4: initially 20% B, linear increase to 36% B within 24 min, followed by rinsing and equilibration steps. Trimer **2** (2*E*,3*E*)-2-[(4-([(1*Z*)-1-carboxy-2-(4-hydroxy-3-methoxyphenyl)eth-1-en-1-yl]oxy)-3-methoxyphenyl)methylidene]-3-[(4-hydroxy-3-methoxyphenyl)methylidene]butanedioic acid) eluted after 24 min. Fraction 5: initially 25% B, linear increase to 33% B within 15 min, followed by rinsing and equilibration steps. 8-8(cyclic)/5-5-TriFA eluted after 14 min. Fraction 6: same conditions as described for fraction 4. The monoethylester of trimer **3** eluted after 16 min. Fraction 8: same conditions as described for fraction 4. Trimer **3** (presumably (2*E*,3*E*)-2-[(3-5-[(1*E*)-2-carboxyeth-1-en-1-yl]-2-hydroxy-3-methoxyphenyl-4-hydroxy-5-methoxyphenyl)methylidene]-3-[(4-hydroxy-3-methoxyphenyl)methylidene]butanedioic acid) eluted after 20 min. Fraction 9: initially 20% C, linear increase to 60% C within 30 min, followed by rinsing and equilibration steps. 8-5(noncyclic)/5-5-TriFA eluted after 28 min and 5-5-DFA eluted after 30.5 min. Fraction 10: initially 20% B, linear increase to 45% B within 20 min, followed by rinsing and equilibration steps. 5-5/8-O-4-TriFA eluted after 20.5 min.

### NMR spectroscopy

Purified compounds were dissolved in 600 μL of acetone-*d*_6_ and analyzed on a Bruker Ascend 500 MHz NMR spectrometer equipped with a Prodigy cryoprobe (Bruker, Rheinstetten, Germany). Spectra from ^1^H, phase-sensitive ^1^H–^13^C HSQC with gradient selection, ^1^H–^13^C HMBC with gradient selection, phase-sensitive ^1^H–^1^H COSY, and phase sensitive ^1^H–^1^H NOESY experiments were acquired at 24.85°C using standard Bruker implementations. Chemical shifts were calibrated against the central acetone residual peak (methyl proton, 2.05 ppm; methyl carbon, 29.84 ppm; Gottlieb et al., 1997).

### Analysis of maize, wheat, sugar beet, and amaranth dietary fiber

Insoluble dietary fiber from maize and wheat kernels, sugar beet pulp, and amaranth seeds were isolated as described earlier (Waterstraat et al., 2016). Briefly, ~20 g of freeze-dried, milled (particle size < 0.5 mm) and defatted plant materials were suspended in phosphate buffer (pH 6.2, 0.08 M, 200 mL), and thermostable α-amylase (1.5 mL, Termamyl 120 L from *Bacillus licheniformis*, 120 KNU/g, Novozymes A/S, Bagsvaerd, Denmark) was added. After incubation with occasional shaking at 92°C for 20 min, samples were cooled to room temperature, and the pH was adjusted to 7.5 with NaOH (0.275 M). Protease (0.6 mL, Alcalase 2.5 L from *B. licheniformis*, 2.5 AU/g, Novozymes A/S) was added, and the suspensions were incubated in a shaking water bath at 60°C for 30 min. After cooling to room temperature, the pH of the suspensions was adjusted to 4.5 using 0.325 M HCl. Samples were incubated with amyloglucosidase (0.7 mL, AMG 300 L from *Aspergillus niger*, 300 AGU/g, Novozymes A/S) with gentle shaking at 60°C for 30 min. Following centrifugation, the residues were washed consecutively with water (60°C, 2 × 100 mL, 1 × 50 mL), 95% ethanol (2 × 100 mL, 1 × 50 mL), and acetone (1 × 100 mL, 1 × 50 mL), and the residues were dried overnight at 60°C.

Saponification of insoluble dietary fiber (up to 100 mg) was performed using 10 mL of 2 M NaOH protected from light and O_2_ with gentle agitation. After 18 h, samples were acidified to pH <2 using concentrated HCl and extracted with diethyl ether. The organic solvent was removed, and the residue was re-dissolved in tetrahydrofuran/H_2_O (1:1, v/v, 500 μL) and analyzed by LC-MS/MS.

The LC-MS/MS system was equipped with a 2690 separations module (pumps, degasser, autosampler and a 996 PDA detector, Waters Corp., Milford, MA, USA), a Micromass Quattro Micro triple-quadrupole mass spectrometer (Waters Corp.), and a column oven (Jetstream Plus, Beckman Coulter). Samples (20 μL) were separated on a phenyl hexyl Luna column (150 × 4.5 mm, 2.6 μm, 100 Å; Phenomenex, Aschaffenburg, Germany). Eluents were formed using H_2_O (A), methanol (B), and acetonitrile (C), each containing 0.01% formic acid. Two different elution gradients were used (flow rate, 0.5 mL/min; column temperature, 23°C) with the first being as follows: initially 70% A, 30% B, linear change to 20% A, 80% B within 59 min, followed by rinsing and equilibration steps. The second elution gradient started with 86% A, 14% C, followed by a linear change to 81% A, 19% C within 7 min, a linear change to 69% A, 9% B, 22% C within 25 min, a linear change to 74% A, 26% C within 1 min, a linear change to 64% A, 36% C within 16 min and final rinsing and equilibration steps. MS parameters were as follows: ionization, ESI negative mode; capillary, 3.0 kV; cone, 28 V; extractor, 2 V; RF lens, 0.1 V; source temperature, 100°C; desolvation gas flow, 700 L/h; cone gas flow, 60 L/h; collision gas pressure, 0.003 mbar; collision energy, 20 eV.

## Results and discussion

### Synthesis

Several methods have been described to synthesize DFA in the past (Ralph et al., 1994, 1998; Yamamoto et al., 1999; Schatz et al., 2006; Lu et al., 2012). In contrast, synthetic routes to obtain TriFA have been sparsely investigated (Bunzel et al., 2008b; Mouterde et al., 2013). Recently, we described the generation of TriFA by radical coupling of ethyl ferulate using the TMEDA complex previously described by Lu et al. (2012) to produce DFA (Waterstraat et al., 2016). Using this approach, purification of individual TriFA from the product mixture is most challenging. Early eluting fractions from the initial fractionation by silica flash chromatography can be purified after saponification by reversed phase RP-HPLC to obtain 8-O-4/8-5(noncyclic)-TriFA, 8-O-4/8-O-4-TriFA, and 8-O-4/8-5(cyclic)-TriFA (Waterstraat et al., 2016). The fraction obtained by rinsing the silica flash chromatography column with ethyl acetate and subsequent saponification, however, contained a large variety of TriFA, dehydrotetraferulic acids, partially saponified oligomers, and additional unknown reaction products. Therefore, an additional clean up step was required prior to the final purification by RP-HPLC. Due to its unique separation mechanism based on size exclusion, hydrophilic and lipophilic interactions, Sephadex LH-20 chromatography was suitable to obtain fractions containing a few compounds only. Sephadex LH-20 chromatography was performed using three elution steps as described for the isolation of oligoferulic acids from maize bran (Figure 1; Bunzel et al., 2004a). Subsequent purification of fractions 5, 9, and 10 by RP-HPLC yielded three TriFA that have been isolated from maize bran before: 8-8(cyclic)/5-5-TriFA (Waterstraat et al., 2016), 8-5(noncyclic)/5-5-TriFA (Bunzel et al., 2006), and 5-5/8-O-4-TriFA (Bunzel et al., 2003a; Rouau et al., 2003). Furthermore, three previously unknown TriFA were purified from fractions 2, 4, and 8, respectively, and structurally elucidated.

### Structural elucidation

The molecular mass of the synthesized and purified compounds **1**, **2**, and **3** was determined to be 578 (quasi molecular ion of *m/z* 577 [M – H]^−^, ESI-MS, negative mode), confirming their trimeric nature.

**Figure 2:**
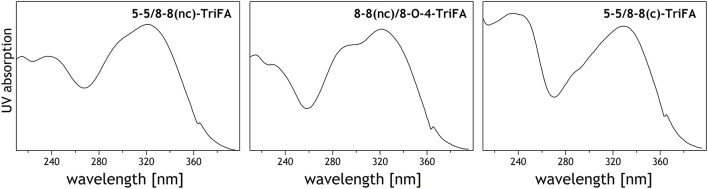
UV spectra of trimer **1** (5-5/8-8(cyclic)-dehydrotriferulic acid (TriFA), trimer **2** (8-8(noncyclic)/8-O-4-TriFA), and trimer **3** (presumably 5-5/8-8(noncyclic)-TriFA). Spikes at 363 nm are not compound specific but caused by the detector used.

UV spectra of these trimers show absorption characteristics that are typical of ferulic acid derivatives (Figure 2); that is, absorption maxima at 320–330 nm, and shoulders between 290 and 305 nm (Dobberstein and Bunzel, 2010). The higher absorption maximum (330 nm) of trimer **1** indicates an 8-8(cyclic)-coupled structural unit, whereas the broader shoulder of trimer **2** suggests an 8-O-4-linkage within the molecule.

**Figure 3:**
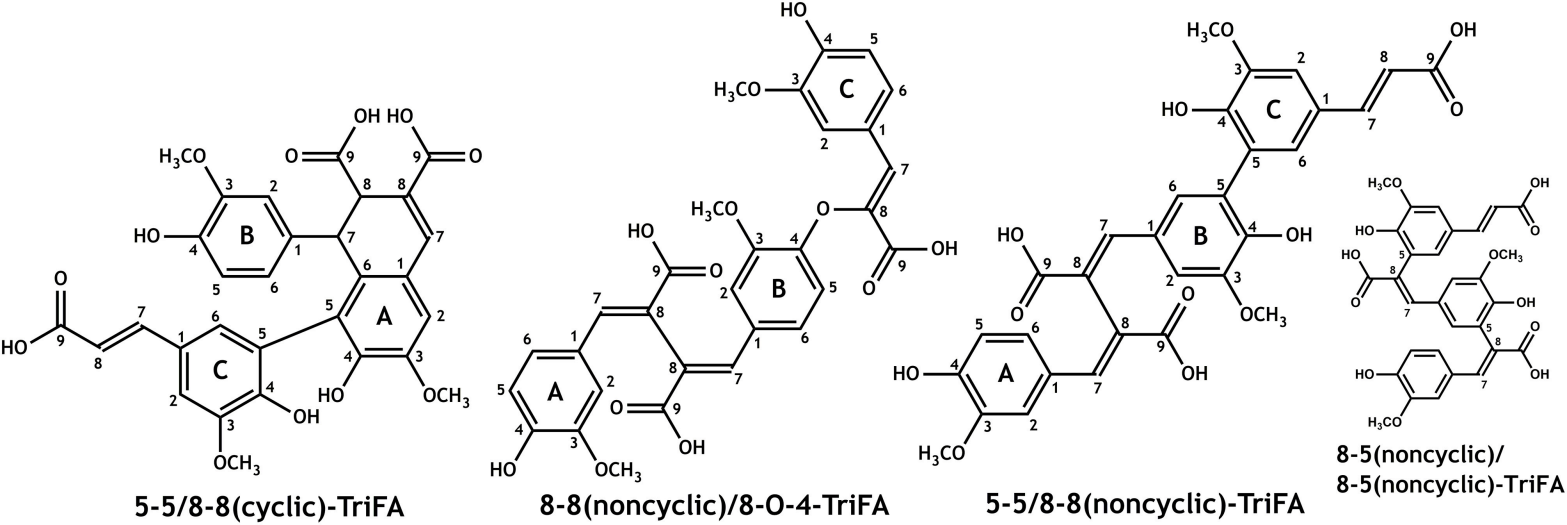
Structures of the novel dehydrotriferulic acids (TriFA) 5-5/8-8(cyclic)-TriFA (trimer **1**), 8-8(noncyclic)/8-O-4-TriFA (trimer **2**), and 5-5/8-8(noncyclic)-TriFA (trimer **3**). Furthermore, the structure of 8-5(noncyclic)/8-5(noncyclic)-TriFA is mentioned in the text.

Spectra from one- and two-dimensional NMR experiments (^1^H, HSQC, COSY, HMBC, and NOESY) were interpreted in order to unambiguously elucidate the structures of trimers **1–3**. The proton spectrum of trimer **1** showed two signals at 3.79 and 4.37 ppm, which are, in combination with the singlet at 7.79 ppm, indicative for an 8-8(cyclic) linkage type (Ralph et al., 1994). Because there was only one more singlet within the aromatic region of the proton spectrum (assigned as A2), it was suggested that position A5 is involved in the linkage to the third ferulic acid unit (Figure 3). Two doublets at 5.98 and 7.26 ppm with coupling constants of 15.9 Hz (representing an unsubstituted *trans*-propenyl side chain) as well as only one doublet of doublets with coupling constants of 8.1 and 2.0 Hz together with a doublet with a coupling constant of 8.1 Hz (representing an unsubstituted proton at position 5) imply that the third ferulic acid unit (C) is coupled via 5-5-linkage to the ferulic acid unit A (Figure 3). All other NMR data are in agreement with the proposed structure of 5-5/8-8(cyclic)-TriFA (Table 1).

**Table 1:** NMR data for trimer **1** [5-5/8-8(cyclic)-dehydrotriferulic acid (TriFA)], trimer **2** [8-8(noncyclic)/8-O-4-TriFA], and trimer **3** [5-5/8-8(noncyclic)-TriFA] measured in acetone-*d*_6_. Carbon chemical shifts were deduced from HSQC and HMBC experiments where possible.

**Trimer 1**			**Trimer 2**			**Trimer 3**		
**Trimer unit**	**^1^H**	**^13^C**	**Trimer unit**	**^1^H**	**^13^C**	**Trimer unit**	**^1^H**	**^13^C**
A1	–	124.2	A1	–	nd	A1	–	128.0
A2	7.21 (1H; s)	112.2	A2	7.29 (1H; d; 2.0)	113.4	A2	7.34 (1H; d; 2.0)	113.4
A3	–	147.9	A3	–	nd	A3	–	148.2
A4	–	147.7	A4	–	nd	A4	–	149.3
A5	–	125.3	A5	6.79 (1H; d; 8.4)^a^	115.9^a^	A5	6.82 (1H; d; 8.2)	115.9
A6	–	130.8	A6	7.13 (1H; dd; 8.4, 2.0)^b^	125.5^b^	A6	7.15 (1H; dd; 8.2, 2.0)	125.5
A7	7.79 (1H; s)	139.1	A7	7.85 (1H; s)^c^	141.7^c^	A7	7.87 (1H; s)	142.5
A8	–	123.1	A8	–	nd	A8	–	126.0
A9	–	168.5	A9	–	168.3	A9	–	168.5
A3-OMe	3.96 (1H; s)	56.2	A3-OMe	3.72 (3H; s)^d^	55.9^d^	A3-OMe	3.75 (3H; s)	55.9
B1	–	136.2	B1	–	nd	B1	–	nd
B2	6.44 (1H; d; 2.0)	112.1	B2	7.44 (1H; d; 2.0)^e^	114.1^e^	B2	7.35 (1H; d; 2.0)	112.1
B3	–	148.2	B3	–	nd	B3	–	148.4
B4	–	146.2	B4	–	148.7	B4	–	147.0
B5	6.62 (1H; d; 8.1)	115.3	B5	6.76 (1H; d; 8.4)^a^	114.0^a^	B5	–	125.7
B6	6.25 (1H; dd; 8.1, 2.0)	120.8	B6	7.11 (1H; dd; 8.4, 2.0)^b^	124.6^b^	B6	7.24 (1H; d; 2.0)	127.9
B7	4.37 (1H, bs)	44.1	B7	7.86 (1H; s)^c^	142.6^c^	B7	7.90 (1H; s)	142.5
B8	3.79 (1H, d; 1.4)	48.1	B8	–	nd	B8	–	126.0
B9	–	173.3	B9	–	168.3	B9	–	168.5
B3-OMe	3.65 (3H; s)	56.1	B3-OMe	3.82 (3H; s)^d^	56.0^d^	B3-OMe	3.79 (3H; s)	56.1
C1	–	126.0	C1	–	nd	C1	–	126.6
C2	7.26 (1H; d; 2.0)	110.2	C2	7.43 (1H; d; 2.0)^e^	113.7^e^	C2	7.34 (1H; d; 2.0)	109.8
C3	–	148.4	C3	–	148.3	C3	–	148.9
C4	–	147.6	C4	–	149.4	C4	–	147.0
C5	–	nd	C5	6.8 (1H; d; 8.3)	115.9	C5	–	125.7
C6	6.17 (1H; d; 2.0)	126.3	C6	7.19 (1H; dd; 8.3, 2.0)	126.0	C6	7.08 (1H; d; 2.0)	126.0
C7	7.26 (1H; d; 15.9)	145.8	C7	7.38 (1H; s)	128.4	C7	7.62 (1H; d; 15.9)	146.0
C8	5.98 (1H; d; 15.9)	115.6	C8	–	138.2	C8	6.43 (1H; d; 15.9)	116.1
C9	–	168.2	C9	–	164.6	C9	–	168.3
C3-OMe	3.96 (3H; s)	56.2	C3-OMe	3.63 (3H; s)	55.6	C3-OMe	3.96 (3H; s)	56.4

*δ in ppm and J in Hz; nd, not determined; superscript letters, assignments may be interchanged (see text)*.

In the proton spectrum of trimer **2**, three signal groups characteristic for unsubstituted ferulate 5-positions (three doublets of doublets with coupling constants of 8 and 2 Hz and three doublets with coupling constants of 8 Hz) were present. Therefore, none of the three ferulate moieties is linked via its 5-position. Indicators for unsubstituted *trans*- (or *cis*-*)*propenyl side chains such as doublets with coupling constants of 16 (or 10) Hz in the aromatic region of the ^1^H spectrum (or respective signals in the COSY spectrum) were absent, demonstrating that all ferulate moieties are linked via their 8-positions. From these data and considering the formation mechanism of ferulic acid oligomers by radical coupling (Ralph et al., 1994), we concluded trimer **2** to be 8-8(noncyclic)/8-O-4-TriFA (Figure 3). All other signals are fully consistent with the proposed structure; however, the assignment of particular signal groups was not fully unambiguous because of overlapping proton chemical shifts. Superscript letters in Table 2 indicate signals that may be interchanged.

**Table 2:** Relative intensities of fragment ions in % measured after fragmentation of the parent ion *m/z* 577.

**Trimer 1**		**Trimer 2**		**Trimer 3**	
***m*/*z***	**Intensity**	***m*/*z***	**Intensity**	***m*/*z***	**Intensity**
533	100	297	100	445	100
489	26.9	163	7.6	489	35.1
577	7.5	209	7.4	430	7.0
445	4.9	282	5.3	533	5.1
409	3.6	137	5.1	413	3.5
365	3.1	445	4.2	415	3.4
321	1.1	161	1.7	294	1.4
		417	1.1		
		146	1.0		

*Trimer **1**, 5-5/8-8(cyclic)-dehydrotriferulic acid (TriFA); trimer **2**, 8-8(noncyclic)/8-O-4-TriFA; trimer **3**, presumably 5-5/8-8(noncyclic)-TriFA*.

Structural elucidation of trimer **3** was more challenging as detailed below. Interpretation of the proton spectrum revealed two doublets with coupling constants of 15.9 Hz indicating the occurrence of one unsubstituted *trans-*propenyl side chain within the molecule. Only one doublet of doublets with coupling constants of 8.2 and 2.0 Hz in combination with one doublet with a coupling constant of 8.2 Hz suggested that two ferulic acid units are coupled via their 5-positions. Moreover, the ^1^H spectrum provided five doublets with coupling constants of 2 Hz, two downfield singlets, and three singlets (with integrals suggesting methyl groups) in the aliphatic region (Table 2). The described proton signals are in agreement with two possible TriFA regioisomers, 5-5/8-8(noncyclic)-TriFA and 8-5(noncyclic)/8-5(noncyclic) (Figure 3). Also, all signals from the two-dimensional NMR experiments performed (HSQC, HMBC, COSY, and NOESY) can reasonably be assigned to both proposed structures. This ambiguity results from the fact that the carbon chemical shifts of both carbons in position 5 and 8 are located between 125 and 126 ppm, regardless of whether the corresponding ferulic acid units are linked via 5-5-, 8-5(noncyclic)-, or 8-8(noncyclic)-linkages (Bunzel et al., 2003a, 2005a, 2006; Funk et al., 2005; Waterstraat et al., 2016). Therefore, additional hints from a second reaction (performed to get ^13^C-labeled trimers) were considered to clarify the chemical structure of trimer **3**.

Using a procedure analogous to the one described in Materials and Methods, [9-^13^C] ferulic acid ethyl ester was radically coupled. The product mixture was fractionated by consecutive flash and Sephadex LH-20 chromatography. Fraction 6 obtained from Sephadex LH-20 chromatography (fraction labeling as shown in Figure 1) contained a [^13^C_3_]TriFA monoethyl ester, which was purified by RP-HPLC and partly subjected to resaponification. As confirmed by LC-DAD-MS/MS analysis, resaponification liberated the ^13^C_3_-labeled trimer **3**, indicating that the ester of trimer **3** was not completely saponified in the first alkaline hydrolysis.

In previous studies it was shown that 8-8(noncyclic)-linked dehydrodisinapates are more resistant against saponification than dehydrodisinapates containing other linkage types (Bunzel et al., 2003b). Also, 8-8(noncyclic)-coupled dehydrodiferulates appear to be more difficult to saponify, as indicated by the purification and unambiguous identification of 8-8(noncyclic)-DFA monoethyl ester from the product mixture described here (data not shown), whereas no other DFA monoethyl esters were detected in significant amounts after saponification of any dimer containing fraction. These findings hint trimer **3** to be 5-5/8-8(noncyclic)-TriFA. Additional information was deduced from NMR data of the purified [^13^C_3_]TriFA monoethyl ester to exclude potential 8-5(noncyclic)/8-5(noncyclic)-coupling of the ferulate units in trimer **3**. All NMR data presented below are discussed based on the assumption that radical coupling of ferulates involving both positions 5 and 8 exclusively results in 8-5(cyclic) structural elements, and that 8-5(noncyclic) structural elements are formed only upon saponification of the ester (Ralph et al., 1994; Bunzel, 2010; Waterstraat et al., 2016). Also, we assume that opening of the cyclic structural units only occurs after or concurrently with the formation of the free acid. Keeping these assumptions in mind, a monomethyl ester of an 8-5/8-5-coupled trimer (as one possible structure of trimer **3**) either contains an 8-5(cyclic)-structural unit, or the ester linkage occurs on the terminal propenylic side-chain that is not involved in the formation of the 8-5(cyclic)-structural units during radical coupling (and therefore in the 8-5(noncyclic) structural units after partial saponification). Otherwise, it can be reasonably excluded that the monomethyl ester of trimer **3** is the ester of an 8-5/8-5-coupled trimer (Figure 3).

**Figure 4:**
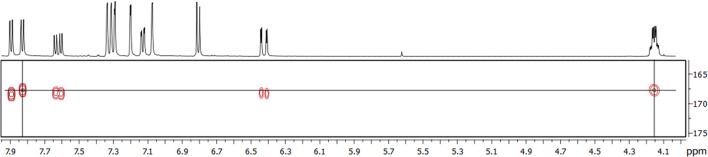
HMBC spectrum of the ^13^C-labeled monoethyl ester of trimer **3**. The horizontal line (δ = 167.7 ppm) indicates a carboxylic ^13^C atom, vertical lines indicate protons at the methylene group (δ = 4.15 ppm) and a proton at position 7 (δ = 7.83 ppm).

All NMR signals of the [^13^C_3_]TriFA monoethyl ester were in accordance with the 5-5/8-8(noncyclic)-linkage pattern of trimer **3**. Additional signals were easily assigned to the methylene and methyl moieties of the ethyl ester. Because signals that are diagnostic for an 8-5(cyclic) structural unit were missing, the existence of this linkage type can be excluded for the monoethyl ester of trimer **3**. Due to the ^13^C-labels in position 9 signals representing protons at positions 7 and 8 showed “additional” coupling constants instead of low-intensity satellites, and signals involving the ^13^C-labels were much more intense in the HMBC spectrum. Interpretation of the HMBC spectrum revealed one carboxylic ^13^C atom that couples with protons from the ethyl group (thereby being the ester) as well as with a proton in position 7 (Figure 4). Because the proton signal of the latter is split into a doublet only [coupling of the proton with the carboxylic ^13^C nucleus (^3^J = 7.4 Hz)], it can be concluded that the ethyl group is attached to a ferulate unit that is substituted in position 8 (thereby not being a terminal propenylic side-chain). Based on these data, it is most unlikely that trimer **3** is 8-5(noncyclic)/8-5(noncyclic)-TriFA, and we tentatively assign trimer **3** as 5-5/8-8(noncyclic)-TriFA.

### Natural occurrence of the previously unidentified trimers

Natural occurrence of the novel TriFA in plant cell walls was demonstrated by analyzing insoluble dietary fiber from monocotyledonous (maize and wheat grain) and dicotyledonous plants (sugar beet pulp and amaranth seeds).

After saponification and extraction of the phenolic acids, the concentrated extracts were analyzed by LC-MS/MS, using two methods differing in the gradient composition of the mobile phase. Using this approach minimizes potential misinterpretation of the data, that is, confusion of matrix compounds with comparable chromatographic and mass spectrometric properties with the trimers of interest. Characteristic fragment ions were determined by fragmentation of trimers **1**, **2**, and **3** (Table 2; parent ion *m/z* 577 [M – H]^−^), and the most intense fragments were chosen as suitable daughter ions for the MS/MS approach: *m/z* 489 and 533 (trimer **1**), *m/z* 297 (trimer **2**), and *m/z* 445, and 489 (trimer **3**).

Using both mobile phase gradients, comparison of retention times with those of the standard compounds of trimers **1**, **2**, and **3** revealed their existence in the alkaline hydrolysate of insoluble dietary fibers from maize grain, wheat grain, sugar beet pulp, and amaranth seeds. Therefore, it can be concluded that these trimers add to the variety of naturally occurring TriFA in the plant cell wall.

## Conclusion

Radical coupling of ethyl ferulate using the copper (II)-TMEDA complex in acetonitrile is a simple and effective method to generate ferulate dimers and trimers. Consecutive fractionation of the reaction mixture and purification of the individual compounds by using flash chromatography, Sephadex LH-20 chromatography, and RP-HPLC yielded nine TriFA in quantities and purities suitable for their use as analytical standard compounds which are mandatory for the development and validation of analytical procedures to analyze ferulate oligomers.

Besides previously described ferulic acid trimers, three novel TriFA were obtained and identified as 5-5/8-8(cyclic)-TriFA (trimer **1**), 8-8(noncyclic)/8-O-4-TriFA (trimer **2**), and, tentatively, 5-5/8-8(noncyclic)-TriFA (trimer **3**). As demonstrated by the analysis of different plant materials, the synthesized trimers also occur naturally in mono- and dicotyledonous plant cell walls, contributing to the complex system of cross-linking cell wall polymers. Therefore, future studies on cell wall cross-links need to include these trimers in the analytical procedures in order to describe plant cell wall cross-linking as detailed and realistic as possible.

## Author contributions

MW and MB designed the research, MW conducted the experiments, MW and MB analyzed the data and results. MW and MB wrote the manuscript. Both authors read and approved the final manuscript.

### Conflict of interest statement

The authors declare that the research was conducted in the absence of any commercial or financial relationships that could be construed as a potential conflict of interest.
